# Comparison of intracorporeal and extracorporeal urinary diversions after laparoscopic radical cystectomy in females with bladder cancer

**DOI:** 10.1186/s12957-019-1678-5

**Published:** 2019-09-12

**Authors:** Liyuan Wu, Feiya Yang, Liming Song, Zejun Xiao, Sujun Han, Song Wu, Sai Liu, Qingbao He, Nianzeng Xing

**Affiliations:** 1 Department of Urology, National Cancer Center/National Clinical Research Center for Cancer/Cancer Hospital, Chinese Academy of Medical Sciences and Peking Union Medical College, No. 17, Panjiayuan South Li, Chaoyang district, Beijing, 100021 People’s Republic of China; 2 Department of Urology, Beijing Chaoyang Hospital, Capital Medical University, No.8 Gongren Tiyuchang Nanlu, Chaoyang district, Beijing, 100020 People’s Republic of China; 3 Department of Urology, Shenzhen Second People’s Hospital, The First Affiliated Hospital of Shenzhen University, No. 3002, Sungang West Road, Futian District, Shenzhen, 518000 People’s Republic of China

**Keywords:** Female, Bladder cancer, Laparoscopic radical cystectomy, Surgical technique

## Abstract

**Purpose:**

To compare the peri-operative outcomes of females undergoing laparoscopic intracorporeal urinary diversions (ICUD) and extracorporeal urinary diversions (ECUD) after laparoscopic radical cystectomies (LRC).

**Patients and methods:**

Thirty-eight females who underwent LRCs and urinary diversions from February 2008 to October 2018 were divided into two groups: the ECUD group (19 patients) and the ICUD group (19 patients). We retrospectively analysed the patients in terms of patients’ demographics, peri-operative outcomes, and oncological follow-ups.

**Results:**

There were significant differences in the mean operative times between ECUDs and ICUDs (364.6 vs. 297.1 min, *p* = 0.007), transfusion rates (37% vs. 5%, *p* = 0.042), time to flatus (5 vs. 3 days, *p* = 0.020), time to ambulation (2 vs. 1 days, *p* = 0.022), and duration of postoperative hospital stays (22 vs. 13 days, *p* = 0.002). The mean lymph node yield was 12.9 in the ECUD group and 18.6 in the ICUD group (*p* = 0.140). Seven out of 19 patients (37%) in the ECUD group and 6 out of 19 patients (32%) in the ICUD group had positive lymph nodes (*p* > 0.9). Two out of 19 ECUD patients (11%) and 4 of 19 ICUD patients (21%) had positive surgical margins (*p* = 0.660). Although there were no differences in major complications at 30 days and in all complications at 90 days, the Clavien grade II complications were significantly different at 30 days (ECUD 8, ICUD 2; *p* = 0.026). The mean follow-up times were 48.7 months (ECUD group) and 26.4 months (ICUD group). There were no statistically significant differences in estimated glomerular filtration rates postoperatively (*p* = 0.516). Seven patients had disease metastases (ECUD 2 out of 19, ICUD 5 out of 19; *p* = 0.405) and 5 died (ECUD 3 out of 19, ICUD 2 out of 19; *p* > 0.9).

**Conclusions:**

ICUDs benefit females by having smaller incisions, faster recoveries, and decreased complication rates.

## Introduction

Primary bladder cancer is a serious worldwide health issue [1], with the male to female morbidity being 3:1 [2]. A radical cystectomy with pelvic lymph node dissection is considered the standard operative treatment for muscle-invasive bladder cancer (MIBC). Compared with open surgery, a laparoscopic radical cystectomy (LRC) is a safe and feasible alternative with fewer complications, reliable pathology and oncologic efficacy, and faster recoveries [3]. Although extracorporeal urinary diversion (ECUD) after LRC has met the standard of care in medical centres for a long time, intracorporeal urinary diversion (ICUD) has gained popularity since the procedure was first described by Gill et al., in 2000 [4]. While traditional surgeries for female patients with MIBC involves anterior exenteration with dissection of the bladder, urethra, uterus, vagina, and both ovaries [5], there is significant controversy regarding which diversion is more beneficial for women after LRC. This article aims to compare ICUDs and ECUDs following LRCs in female patients by analysing their demographics, peri-operative outcomes, and oncological follow-ups.

## Materials and methods

### Patients

From February 2008 to October 2018, 38 female patients with bladder cancer received LRCs with urinary diversions, of whom 19 patients underwent LRCs with ECUDs and 19 underwent LRCs with ICUDs. All operations were performed by an experienced surgeon in a single centre. Indications included muscle invasive bladder cancer, T1G3 and recurrent superficial bladder cancer, or extensive non-muscle-invasive bladder cancer (NMIBC) that could not be controlled by transurethral resection bladder cancer and intravesical treatments. All patients received pelvic magnetic resonance imaging or enhanced computed tomography examinations before their surgeries. No distant metastases were identified on bone scans, chest X-rays, and sonographies. The Clavien-Dindo classification was used to assess postoperative complications [6].

### Surgical technique

Under intravenous anaesthesia, patients were placed in lithotomy positions. We used a five-port transperitoneal approach. The first 10-mm trocar (for the camera) was placed at the 2–4 cm upper level of the umbilicus. Two 12-mm trocars were placed at the bilateral external rectus line, and two 5-mm trocars were placed at the 3-cm interior bilateral anterior superior iliac spines, at the same level as the first trocar.

#### Anterior pelvic exenteration

Laparoscopic dissection began with generous mobilisation of the suspensory ligament of the ovary. The suspensory ligaments were transected with Ligasure^TM^ (Valleylab, USA). The bilateral ureters were then divided at the common iliac artery bifurcation and the umbilical arteries were dissected using Hem-O-Lok clips. The broad ligaments of the uterus were disassociated along the pelvic wall and the cardinal ligament of the uterus was cut off. The fornix of the vagina was incised transversely until reaching the posterior urethra, after lifting the uterus and bilateral attachments using forceps. Subsequently, the endopelvic fascia was dissected at both sides, and the dorsal vein complex (DVC) was ligated with 2-0 V-Loc sutures to avoid using instruments that caused heat damage, thereby preserving sexual function. The anterior urethra was separated along the bladder neck and removed using scissors in a ‘cone shape’ to protect the internal urethral sphincter. Hem-O-Lok clips were used to close the urethra. The ureters were also closed using Hem-O-Lok clips (two towards the bladder and one towards the proximal end) and were then transected down the middle using scissors. Frozen section examinations of the ends of the urethra and the distal ureter were subsequently performed. The bladder and the female reproductive organs were placed into an EndoCatch bag and were removed together through the vagina, which was later closed with a running suture.

#### Pelvic lymph node dissection

The external iliac artery sheath was cut from the iliac artery branch to the distal end. Dissections of the internal iliac lymph nodes, pre-sacral lymph nodes, obturator fossa lymph nodes, and external iliac lymph nodes were then performed. Distinct area lymph nodes were marked and removed for pathological examination.

#### Urinary diversion

For ECUDs, the ileum was extracted through the 10–15-cm incision below the umbilicus. For ileal conduits [7], a 15-cm-long ileal segment was isolated about 20 cm proximally to the ileocaecum; for orthotopic ileal neobladders [8], we choose a 60-cm-long ileal segment. The ileal segments were detubularised along their anti-mesenteric border to form a new reservoir outside the abdominal cavity. Before the suturing of the new reservoir was complete, both ureters were spatulated for approximately 3 cm and Mono-J ureteric stents (6F) were inserted in both ureters. The ureters were then anastomosed with the proximal end of reservoir end-to-end. Finally, a circular disc of skin was excised at the right 12-mm port, and the outflow limb was fixed with fascia and skin. For ICUDs, the above urinary diversion was completed in the abdominal cavity. Nineteen patients underwent ECUD and 19 patients underwent ICUD.

### Statistical analysis

Continuous data were analysed using mean values with standard deviations and compared by Student’s *t* tests. Categorical data were analysed using medians and ranges and compared with the test. *p* values < 0.05 were considered statistically significant. Kaplan-Meier analysis was used to calculate survival probabilities. All statistics were performed using SPSS v. 21 (IBM Corp, Armonk, NY, USA).

## Results

There were no significant differences in baseline characteristics, body mass indices, Charlson comorbidity indices, American Society of Anaesthesiologists scores, pre-creatinine, and pre-operative stage distributions, etc. (Table 1).

**Table 1:** Patients demographics

Variables	Extracorporeal	Intracorporeal	*P*
Patients, *n*	19	19	
Age (mean ± SD), year	62.5 ± 8.54	67.9 ± 11.40	0.173
BMI (mean ± SD), kg/m^2^	25.5 ± 2.97	25.3 ± 2.74	0.867
Charlson comorbidity index (median [range])	4 [2–9]	3 [2–5]	0.548
ASA score, *n*			> 0.9
1–2	17 (89%)	17 (89%)	
3	2 (11%)	2 (11%)	
Neoadjuvant chemotherapy, n (%)	4 (21%)	3 (16%)	> 0.9
Pre-eGFR (mean ± SD), mL/(min·1.73 m^2^)	86.3 ± 28.87	85.9 ± 34.57	0.972
Pre-Hb (mean ± SD), g/L	116.9 ± 16.81	118.6 ± 13.94	0.765
Preoperative diagnosis, *n*			> 0.9
NMIBC	7 (37%)	8 (42%)	
MIBC	12 (63%)	11 (58%)	
Clinical stage			0.720
T_a_/T_1_/T_is_	7 (37%)	8 (42%)	
T_2_	9 (47%)	5 (26%)	
T_3_	3 (16%)	5 (26%)	
T_4_	0 (0%)	1 (5%)	
Clinical stage			0.405
N_0_	17 (89%)	14 (74%)	
N_1_	2 (11%)	5 (26%)	

*BMI* body mass index, *ASA* American Society of Anesthesiologists, *Pre-eGFR* pre-estimated glomerular filtration rate, *MIBC* muscle-invasive bladder cancer, *NMIBC* non-muscle-invasive bladder cancer

A total of 38 patients underwent LRCs with ICUDs or ECUDs: 19 had ECUDs (11 conduits and 8 neobladders), and 19 had ICUDs (13 conduits and 6 neobladders) (Table 2). There were significant differences in ECUDs and ICUDs for mean operative times (364.6 ± 72.30 vs. 297.1 ± 48.24 min, *p* = 0.007), transfusion rates (37% vs. 5%, *p* = 0.042), time to flatus (5 vs. 3 days, *p* = 0.020), time to ambulation (2 vs. 1 days, *p* = 0.022), and duration of postoperative hospital stays (22 vs. 13 days, *p* = 0.002). However, there were no statistical differences in estimated blood loss (EBL) (393.1 ± 387.46 vs. 267.0 ± 205.69, *p* = 0.252), intensive care unit (ICU) stay rates post-surgery (10% vs. 5%, *p* > 0.9), onset of liquid diets (5 vs. 4 days, *p* = 0.087), and length of time before Jackson-Pratt drains were removed (14 vs. 10 days, *p* = 0.549). Overall, there were no significant differences in pathologic stages (*p* = 0.527). Mean lymph node yield was 12.9 in the ECUD group and 18.6 in the ICUD group (*p* = 0.140). Seven out of 19 patients (37%) in the ECUD group and 6 out of 19 patients (32%) in the ICUD group had positive lymph nodes (*p* > 0.9). Two out of 19 patients in the ECUD group (11%) and 4 of 19 patients in the ICUD group (21%) had positive surgical margins (*p* = 0.660).

**Table 2:** Surgical outcomes for female patients

Variables	Extracorporeal (*n* = 19)	Intracorporeal (*n* = 19)	*P*
Diversion type, *n* (%)			0.737
Ileal conduit	11 (58%)	13 (68%)	
Neobladder	8 (42%)	6 (32%)	
Operative time (mean ± SD), min	364.6 ± 72.30	297.1 ± 48.24	0.007
EBL (mean ± SD), mL	393.1 ± 387.46	267.0 ± 205.69	0.252
Transfusion, *n* (%)	7 (37%)	1 (5%)	0.042
ICU stay after surgery, n (%)	2 (10%)	1 (5%)	> 0.9
Time to flatus (median [range]), days	5 [3–9]	3 [1–6]	0.020
Time to intake of liquid diet (median [range]), days	5 [4–8]	4 [2–18]	0.087
Time to ambulation (median [range]), days	2 [0–5]	1 [1–3]	0.022
Time to remove Jackson-Pratt drain (median [range]), days	14 [5–23]	10 [4–30]	0.549
Length of hospital stay after surgery (median [range]), days	22 [14–45]	13 [7–22]	0.002
Final pathologic stage			0.527
T_a_/T_1_/T_is_	10 (53%)	9 (47%)	
T_2_	6 (32%)	4 (21%)	
T_3_	2 (10%)	5 (26%)	
T_4_	1 (5%)	1 (5%)	
Pathologic stage			0.631
N_0_	13 (68%)	13 (68%)	
N_1_	3 (16%)	0 (0%)	
N_2_	3 (16%)	3 (16%)	
N_3_	0 (0%)	3 (16%)	
Lymph node yield (mean ± SD), *n*	12.9 ± 7.81	18.6 ± 11.51	0.140
Lymph node positive patients, *n* (%)	7 (37%)	6 (32%)	> 0.9
Positive surgical margin, *n* (%)	2 (11%)	4 (21%)	0.660

*EBL* estimated blood loss

For ileal conduits, there were statistical differences between ECUD and ICUD transfusion rates (46% vs. 7.7%, *p* = 0.048); however, there were no statistical differences in the mean operative times (330.0 ± 53.67 vs. 288.5 ± 45.99 min, *p* = 0.101), EBLs (600.0 ± 485.80 vs. 242.7 ± 178.75, *p* = 0.134), postoperative ICU stay rates (18% vs. 8%, *p* = 0.435), time to flatus (4 vs. 3 days, *p* = 0.099), onset of liquid diets (5 vs. 4 days, *p* = 0.169), time to ambulation (1 vs. 1 days, *p* = 0.052), time before removal of the Jackson-Pratt drains (12 vs. 8 days, *p* = 0.758), and duration of postoperative hospital stays (15 vs. 14 days, *p* = 0.234) (Table 3). For neobladder reconstructions, there were statistical differences in the duration of postoperative hospital stays (23 days [ECUD] vs. 10 days [ICUD], *p* = 0.039). There were no statistical differences in any other categories (Table 4).

**Table 3:** Surgical outcomes for ileal conduit

Variables	Extracorporeal (*n* = 11)	Intracorporeal (*n* = 13)	*P*
Operative time (mean ± SD), min	330.0 ± 53.67	288.5 ± 45.99	0.101
EBL (mean ± SD), mL	600.0 ± 485.80	242.7 ± 178.75	0.134
Transfusion, *n* (%)	5 (46%)	1 (7.7%)	0.048
ICU stay after surgery, *n* (%)	2 (18%)	1 (8%)	0.435
Time to flatus (median [range]), days	4 [3–7]	3 [1–6]	0.099
Time to intake of liquid diet (median [range]), days	5 [4–8]	4 [2–18]	0.169
Time to ambulation (median [range]), days	1 [0–2]	1 [1–3]	0.052
Time to remove Jackson-Pratt drain (median [range]), days	12 [7–17]	8 [4–30]	0.758
Length of hospital stay after surgery (median [range]), days	15 [14–23]	14 [7–22]	0.234
Lymph node yield (mean ± SD), *n*	9.8 ± 10.15	20.2 ± 11.08	0.070
Lymph node positive patients, *n* (%)	5 (46%)	5 (39%)	0.527
Positive surgical margin, *n* (%)	2 (18%)	3 (23%)	0.585

**Table 4:** Surgical outcomes for neobladder

Variables	Extracorporeal (*n* = 8)	Intracorporeal (*n* = 6)	*P*
Operative time (mean ± SD), min	394.3 ± 76.35	352.5 ± 10.61	0.486
EBL (mean ± SD), mL	230.0 ± 158.01	425.0 ± 388.91	0.606
Transfusion, *n* (%)	2 (25%)	0 (0%)	0.308
ICU stay after surgery, *n* (%)	0 (0%)	0 (0%)	.
Time to flatus (median [range]), days	5 [3–9]	2 [3–5]	0.361
Time to intake of liquid diet (median [range]), days	5 [4–6]	4 [3–5]	0.280
Time to ambulation (median [range]), days	2 [1–5]	2 [2–3]	0.190
Time to remove Jackson-Pratt drain (median [range]), days	16 [5–23]	18 [8–28]	0.769
Length of hospital stay after surgery (median [range]), days	23 [20–45]	10 [7–15]	0.039
Lymph node yield (mean ± SD), *n*	15.4 ± 4.39	8.5 ± 12.02	0.562
Lymph node positive patients, *n* (%)	2 (25%)	1 (17%)	0.615
Positive surgical margin, *n* (%)	0 (0%)	1 (17%)	0.429
Complete continence in the daytime, *n* (%)	6 (75%)	6 (100%)	0.308
Complete continence at night, *n* (%)	6 (75%)	3 (50%)	0.343

Overall, there were no statistical differences in the rates of minor and major 30 days or 90 days complications (Table 5). In the ECUD group, 10 patients (52%) presented with at least one complication within 30 days, and 9 of these patients (47%) had minor complications (grades 1–2). Similarly, 6 patients (37%) in the ICUD group presented with at least one complication and 1 (5%) had a Clavien grade V complication who later died due to multiple organ failure resulting from a myocardial infarction within 30 days. Interestingly, there were significant differences at Clavien grade II (ECUD 8, ICUD 2; *p* = 0.026) within 30 days. The most common complications in the ECUD group were haematological diseases, while infections were the most common in the ICUD group (Tables 6 and 7).

**Table 5:** Follow-up data

Variables	Extracorporeal (*n* = 19)	Intracorporeal (*n* = 19)	*P*
Follow-up time (mean ± SD), months	48.7 ± 30.58	26.4 ± 8.29	0.329
Post- eGFR (mean ± SD), mL/(min·1.73 m^2^)	79.0 ± 19.95	86.2 ± 34.7	0.516
30-day complication rates, *n* (%)			0.227
Minor (I–II)	9 (47%)	5 (32%)	0.313
Major (III–V)	1 (5%)	1 (5%)	> 0.9
90-day complication rates, *n* (%)			0.142
Minor (I–II)	1 (5%)	2 (11%)	> 0.9
Major (III–V)	0 (0%)	2 (11%)	0.486
Metastasis, *n* (%)	2 (11%)	5 (26%)	0.405
Overall survival, *n* (%)	16 (84%)	17 (89%)	> 0.9

*Post-eGFR* post-estimated glomerular filtration rate

**Table 6:** Complications classified by Clavien grade

Clavien grade	30 days			90 days		
	ECUD	ICUD	*P*	ECUD	ICUD	*P*
1	1	3	0.302	0	1	> 0.9
2	8	2	0.026	1	1	> 0.9
3	1	0	> 0.9	0	1	> 0.9
4	0	0		0	0	
5	0	1	> 0.9	0	1	> 0.9

**Table 7:** Complications by system

	ECUD		ICUD	
	0–30 days	30–90 days	0–30 days	30–90 days
Haematological (*n* = 11)				
Hypoproteinemia	8	1	0	0
Great saphenous varicosity	1	0	0	0
Deep venous thrombosis	0	0	1	0
Infectious (*n* = 5)				
Wound infection	0	0	1	0
Pneumonia	1	0	0	1
Vaginitis	0	0	2	0
Gastrointestinal (*n* = 3)				
Ileus	0	0	1	2
Multiple organ failure (*n* = 1)	0	0	1	0
Pulmonary metastasis (*n* = 1)	0	0	0	1

### Follow-up data

**Fig. 1:**
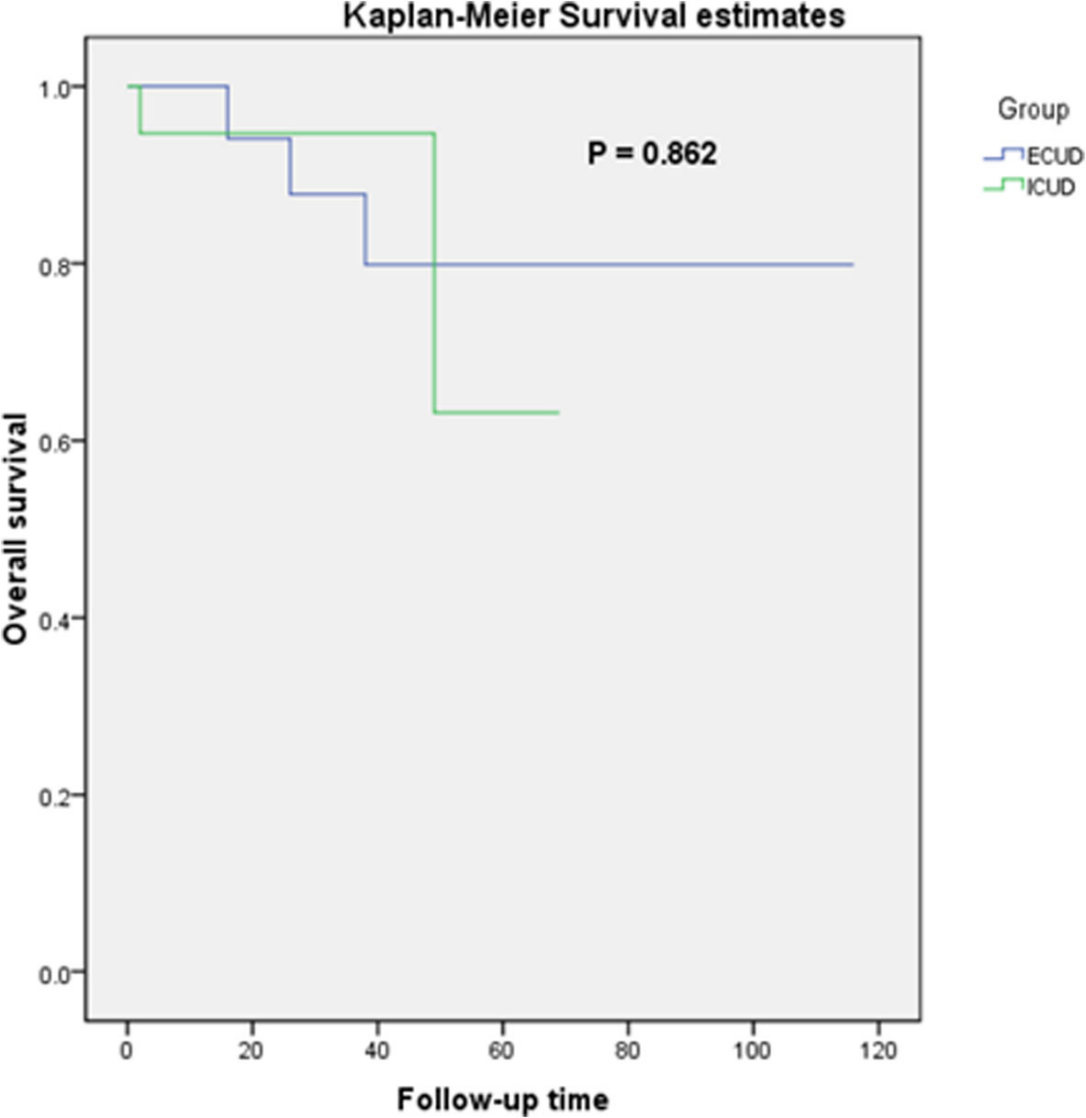
Kaplan-Meier survival estimates between the ECUD and ICUD groups. Log-rank test *p* = 0.862

The mean follow-up times were 48.7 and 26.4 months for the ECUD and ICUD groups, respectively. There were no statistically significant differences in the estimated glomerular filtration rate postoperatively (*p* = 0.516). Seven patients had disease metastases (ECUD 2 out of 19, ICUD 5 out of 19; *p* = 0.405) and 5 died (ECUD 3 out of 19, ICUD 2 out of 19; *p* > 0.9). There was no recurrence. There was no statistical differences in oncologic outcomes (recurrence, metastasis, or overall mortality) (Table 5) or Kaplan-Meier analyses between the two groups (Fig. 1).

## Discussion

An LRC with an ICUD is technically challenging and requires extensive operative experience. The procedure has seldomly been reported since the first two cases reported by Gill et al. [9]. Recently, with the popularisation and improvement of laparoscopic techniques, LRCs and urinary diversions have become more popular in female patients, although females, with their distinctive pelvic anatomies, have many surgical differences in radical cystectomies compared with males. While limited reports on laparoscopic radical cystectomies in females have begun to emerge, this is the first paper comparing intracorporeal and extracorporeal urinary diversions after LRCs in women. These operations were performed entirely by one surgeon in a single centre.

Hussein et al. [10] retrospectively evaluated the outcomes of robot-assisted radical cystectomies with ICUDs (*n* = 1094) and ECUDs (*n* = 1031) from 26 institutions since 2005. Patients having ICUDs showed shorter operative times (357 min vs. 400 min, *p* < 0.001), less blood loss (300 mL vs. 350 mL, *p* < 0.001), and fewer received blood transfusions (5% vs. 13%, *p* < 0.001), suggesting that the complete laparoscopic approach was technically more efficient. Similarly, in our study, the mean operative time between the ECUD and ICUD groups (364.6 ± 72.30 vs. 297.1 ± 48.24 min, *p* = 0.007), transfusion rates (37% vs. 5%, *p* = 0.042), and EBLs (400.8 mL vs. 267.0 mL, *p* = 0.252) showed advantages for ICUDs to some extent. The time to flatus (3 days, *p* = 0.020), ambulation (1 days, *p* = 0.022), and durations of hospital stays (13 days, *p* = 0.002) postoperatively in the ICUD group were shorter than in the ECUD group. It was suggested that ICUDs were also associated with quicker recoveries. This may be partially due to decreased intestinal exposures, shorter operative times, and smaller EBLs. Additionally, enhanced recovery programmes, which we began in 2014 [11], were identified as an important part of postoperative treatments for radical cystectomies [12] that may have resulted in faster patient recoveries in the ICUD group in our study.

Ahmed et al. reported on 935 patients in 18 international centres who had undergone ICUDs or ECUDs following robot-assisted radical cystectomies resulting in 43% in the ECUD group and 35% in the ICUD group having complications within 30 days of surgery (*p* = 0.07). The 90-day complication rate was not significant between the two groups (ICUD 41% vs. ECUD 49%, *p* = 0.055) [13]. In our study, Clavien grade II complications were significantly different at 30 days (ECUD 8, ICUD 2; *p* = 0.026) potentially indicating that the ICUD group was associated with decreased complication rates. Azzouni et al. studied 81 patients after robot-assisted intracorporeal ileal conduits (overall complication rate, 81%) and reported postoperative complications in the first 90 days, 15 patients of whom had high-grade complications (15%). A total of 164 complications were recorded with the most common complication being urinary tract infections [14]. Infections were also more common in the ICUD group in our study, with four patients having postoperative complications within 90 days (22%) and two patients having high-grade complications (11%). This indicates that our surgical technique could have had some benefits in terms of reducing postoperative complications and that robot-assisted intracorporeal ileal conduits are not better than laparoscopies for postoperative complications within 90 days.

In this group, orthotopic ileal neobladder reconstructions were performed in 14 cases, all of whom could urinate on their own 12 months postoperatively without catheterisation. The complete daytime continence rate was 88.9% and 77.8% at night. Orthotopic ileal neobladder reconstructions can maximally imitate the natural urination function and significantly improve the patients’ psychological acceptance of surgery. To some extent, it might ameliorate the postoperative quality of life. Our experience regarding orthotopic ileal neobladder reconstructions are as follows: (1) the procedure should comply with the principles of tumour resection, and the urethral autonomic nerve should be preserved to the greatest extent; (2) the bladder neck should be separated close to the bladder wall; and (3) the bilateral tissue of the anterior vaginal wall should be preserved. Overall, the patients were satisfied with their postoperative urinary continence based on the follow-up results.

This study also had several limitations. First, the sample size was relatively small; thus, it was difficult to arrive at a definitive conclusion. Secondly, this was a cohort study and not a randomised trial; therefore, there were selection biases and limited information.

## Conclusion

ICUD after LRC in women is technically feasible. Understanding the female pelvic anatomy and mastering the techniques are helpful to optimise surgical procedures and greatly decrease complications. Compared with ECUDs, ICUDs are potentially beneficial due to smaller incisions, faster recoveries, and decreased complication rates.

## Data Availability

The datasets supporting the conclusions of this article are included within the article.

## Abbreviations

EBL: Estimated blood loss
ECUD: Extracorporeal urinary diversion
ICUD: Intracorporeal urinary diversion
LRC: Laparoscopic radical cystectomy
MIBC: Muscle-invasive bladder cancer
NMIBC: Non-muscle-invasive bladder cancer
